# Evaluation of the Efficacy of an Aflatoxin Binder in Holstein Dairy Cows in Perú Under Field Conditions

**DOI:** 10.3390/toxins18090386

**Published:** 2026-09-07

**Authors:** Ivonne Salazar-Rodríguez, Iván López, Patricia Glorio-Paulet, Isabel Cristina Molina-Botero, Arturo Gerardo Valdivia-Flores, Carlos Gómez-Bravo

**Affiliations:** 1 Facultad de Zootecnia, Universidad Nacional Agraria La Molina, Av. La Molina S/N, La Molina, Lima 12, Peru; i.molina@cgiar.org (I.C.M.-B.); cagomez@lamolina.edu.pe (C.G.-B.); 2 Facultad de Zootecnia, Universidad Nacional Agraria La Molina, Establo Jetepeque, La Libertad, Av. La Molina S/N, La Molina, Lima 12, Peru; ilopez@pdandina.com.pe; 3 Facultad de Industrias Alimentarias, Universidad Nacional Agraria La Molina, Av. La Molina S/N, La Molina, Lima 12, Peru; pgp@lamolina.edu.pe; 4 Tropical Forages Program of the International Center for Tropical Agriculture (CIAT), Km 17 Palmira, Valle del Cauca 763022, Colombia; 5 Agricultural Sciences Centre, Universidad Autónoma de Aguascalientes, Av. Universidad 940, Aguascalientes 20100, Mexico

**Keywords:** aflatoxin M1, mycotoxins, *Aspergillus*, transfer rate, carry-over rate, animal health, dairy farming feeds, dairy production, organic adsorbents

## Abstract

The strategy of adding mycotoxin binders (MTBs) to feed lactating cows to reduce the absorption of aflatoxins (AFs) in the gastrointestinal tract and reduce milk contamination by hydroxylated AF compounds, especially AFM1, to below the maximum contaminant level (MCL) has become widespread. However, the indiscriminate use of MTBs is very common. The objective of the study was to determine, under field conditions, the efficacy of an MTB in reducing AFM1 contamination in cow’s milk. Feed and raw milk were evaluated on-farm using lateral flow immunochromatography devices. In a 2 × 2 Latin square design, in two separate test periods, two diets were administered consecutively to each of twelve Holstein cows (multiparous, 220 ± 20 lactation days): 1: Basal diet naturally contaminated (120 AFB1 µg/d); 2: Basal diet plus an MTB (calcium carbonate and yeast fragments; 150 g/d). MTB intake effectively reduced the mean concentration, daily excretion, and percentage transfer rate of AFM1 in milk (0.724 ± 0.045 to 0.479 ± 0.055 µg/kg, *p* < 0.01; 15.6 ± 1.7 to 9.7 ± 1.5 µg/d, *p* < 0.05; and 13.0 ± 1.4 to 8.1 ± 1.3%, *p* < 0.05, respectively). These results highlight the need to evaluate, under field conditions, the effectiveness of MTBs as part of a mycotoxin contamination control program on dairy farms.

## 1. Introduction

Mycotoxins (MTs) are low-molecular-weight secondary metabolites; they are produced by various soil fungi or molds and serve as a defense mechanism against other organisms that also consume vegetable substrates. The most dangerous MTs are aflatoxins (AFs), which are produced by the genus *Aspergillus*, particularly *A. flavus*, *A. parasiticus*, and *A. nomius*. AFs possess significant toxic, mutagenic, carcinogenic, and immunosuppressive properties [1,2,3].

The AFs present in agricultural crops are identified as AFB1, AFB2, AFG1, and AFG2, of which the first is the most abundant and the most toxic [4,5]. When contaminated feed is ingested by lactating cows, AFs are absorbed in the gastrointestinal tract and rapidly reach their peak concentration (35 min); subsequently, the AFs are metabolized by the liver’s mixed-function oxidase system in dairy cows, causing their blood concentration to decrease by half every 15 h in a first-order elimination process. This metabolic process forms oxidizing compounds that bind to cellular structures, impairing their function and altering the expression of nucleic acids [6]. When AFB1 is hydroxylated in the liver, a derivative called aflatoxin M1 (AFM1) is formed, which can be excreted into milk one to two hours after the contaminated feed is eaten and continues to be excreted for up to 96 h [6,7,8,9,10]. AFM1 is not eliminated by heat treatments or pasteurization; furthermore, exposure to AFM1, even at low concentrations, poses a serious risk to human health, especially for children and older adults [11,12].

The clinical signs of aflatoxin poisoning in cattle depend on the dose and duration of exposure; in acute aflatoxicosis, fatty liver and impaired coagulation occur in cattle exposed to high levels of aflatoxins (1.0–33.5 mg/kg) [5,13]. Chronic aflatoxicosis (0.1–0.8 mg/kg), on the other hand, is typically subclinical and unrelated to the timing of AF exposure [13,14], although it causes a significant decline in the animals’ productive and reproductive performance, as well as milk contamination by AFM1 [4,15].

It is estimated that mycotoxins contaminate between 60 and 80 percent of the world’s crops and agricultural products, particularly cereals, seeds, and oilseeds [16]. Consequently, various legal, physical, chemical, and biological strategies have been established to control contamination by MTs; one of the most common strategies involves setting maximum permitted concentrations of MTs in food and feed within a region or country, called action levels or maximum contaminant levels (MCLs). European legislation establishes MCLs for AFs in dairy cattle feed and milk (5.0 µg/kg and 0.05 µg/kg); however, in several American and Asian countries, higher MCLs are established [17,18,19]. The monitoring of food and feed contamination by AFs and other MTs is conducted voluntarily in the Peruvian context both by government agencies and by agro-industrial companies to meet the MCLs recommended by the Codex Alimentarius for AFB1 and AFM1 (20.0 and 0.5 µg/kg) [20].

Another very common strategy among farmers is the use of feed additives to inhibit or reduce the absorption of mycotoxins, promote their excretion, or modify their mode of action [21]. These mycotoxin binders (MTBs) act at the ruminal level by covalently binding to MTs, reducing intestinal absorption, promoting their excretion in feces, and reducing the presence of AFM1 in milk; the most common forms of MTBs are clays, organic adsorbents, and yeast-derived polysaccharides [21,22,23,24,25].

The ease with which MTBs can be incorporated into diets as a mechanism for controlling MT contamination in feed has boosted global supply to over 1.25 million metric tons in 2023, with an annual growth rate of 8% [26]. However, dairy farmers’ choice of a specific commercial product is based almost exclusively on marketing criteria and industry reports regarding contamination of their products. Consequently, farmers are often unsure of the actual efficacy of MTBs, the factors influencing their outcomes, or the need to implement complementary measures to prevent contamination of crops and stored grain. Therefore, the objective of the present study was to determine, under field conditions, the effectiveness of an MTB, based on calcium carbonate and yeast fragments, in significantly reducing aflatoxin M1 concentration in raw cow’s milk.

## 2. Results

### 2.1. Aflatoxin B1 in Feedstuffs

The dairy cows included in the study were fed a basal diet (BD) at a daily amount of 40.2 kg. The total daily AFB1 intake per cow was estimated at 120 µg of AFB1, derived mainly from contaminated corn (86.7%; Table 1), while the other ingredients contributed only 13.3% of the AFB1 in the diet. Although the average concentration of AFB1 in this diet indicated a level of contamination (adjusting ND data to half LOD: 3.0 µg/kg; excluding ND data: 2.6 µg/kg) below the MCL recommended in the Peruvian region for feeding lactating cows (20.0 µg/kg), the local dairy industry reported excessive contamination of milk with AFM1 (≥0.5 µg/kg) [19,20]. The diet of the cows with the highest milk production in the herd was occasionally supplemented with an MTB (calcium carbonate and yeast fragments, 150 g/cow), which was sometimes added to the premix of sodium chloride, bicarbonate, and monensin sodium; in those cases, the MTB was used without knowing whether MTs were present in the feed and without evaluating the product’s actual effectiveness. Therefore, the efficacy of adding MTBs to the basal diet (BD+MTB) was evaluated by comparing it to the BD alone; this controlled trial also used a control diet (CD) made with ingredients that did not contain detectable levels of AFB1.

### 2.2. Reduction in Milk AFM1 Content

**Figure 1:**
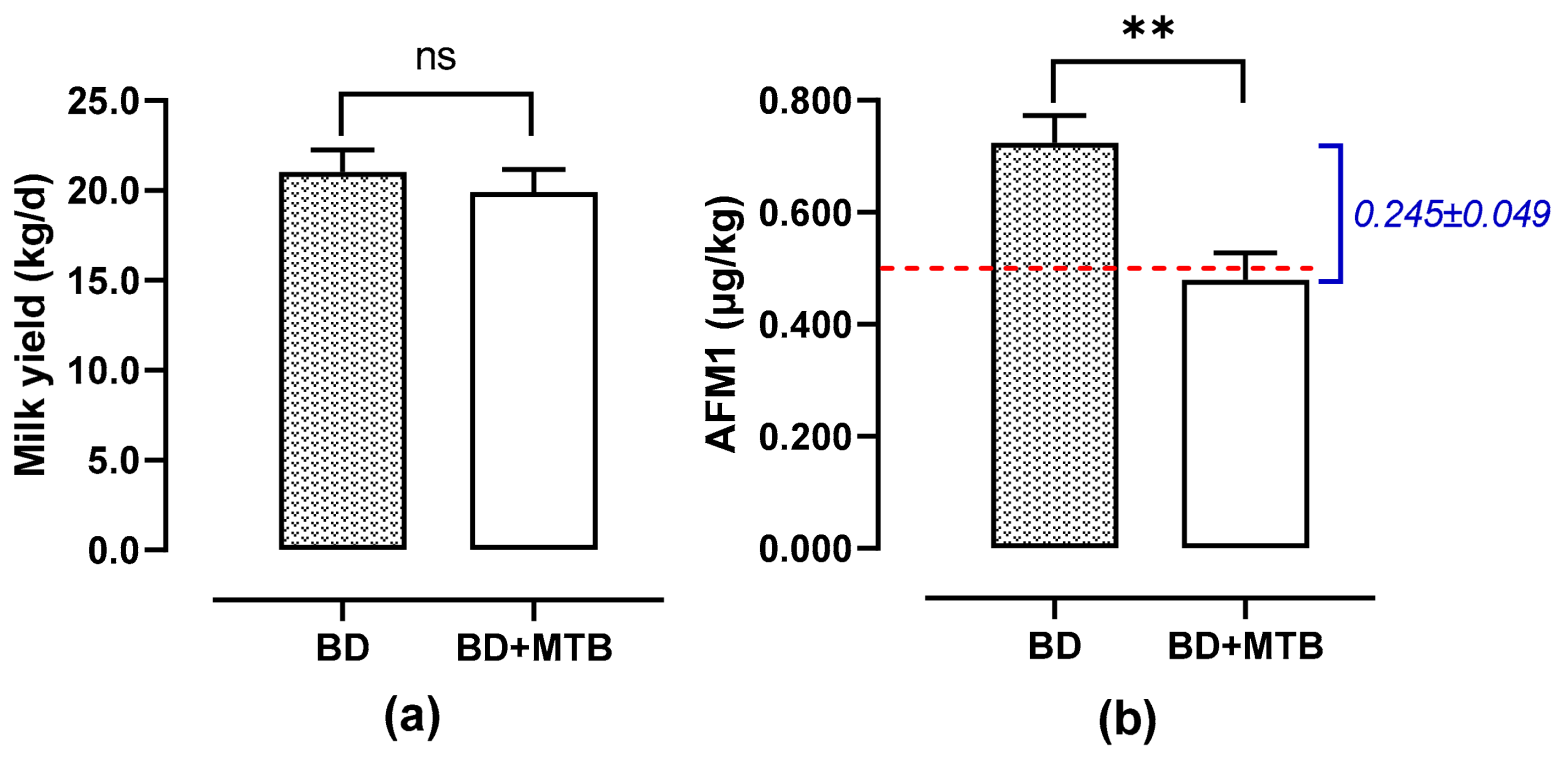
Least squares means (±SEM) of milk production and aflatoxin M1 (AFM1) content in the milk of dairy cows fed either a basal diet (BD) or a basal diet plus a mycotoxin binder (BD+MTB). (**a**) Average daily milk production per cow; (**b**) AFM1 concentration in milk. The difference between the means (blue text), applicable maximum contaminant limit (dashed red line) and Tukey’s HSD test *p*-value are also indicated (ns = not significant, ** *p* < 0.01).

Cows fed the basal diet plus the MTB showed no significant change in daily milk production (19.9 ± 1.3; 95% CI: 17.4–22.5; *p* > 0.05) compared with those fed the basal diet alone (21.0 ± 1.1; 95% CI: 18.5–23.6; Figure 1a). The average concentration of AFM1 in the milk of cows fed the basal diet was estimated at 0.724 ± 0.045 µg/kg (95% CI: 0.623–0.826; Figure 1b). Furthermore, the inclusion of the MTB agent based on calcium carbonate and yeast cell walls reduced the mean AFM1 concentration in the total milk production of this group of cows (*p* < 0.01), which reached a significantly lower concentration (0.479 ± 0.055; 95% CI: 0.378–0.581 µg/kg). These results suggest that MTB added to the BD was 33.8% effective in reducing the mean concentration of AFM1 in raw milk (*p* < 0.01).

In the BD group, 91.7% (n = 11/12) of the samples had AFM1 concentrations above the MCL recommended in the Peruvian context, while in the BD+MTB group, 58.3% (n = 7/12) of the milk samples were below that limit; however, the differences were not statistically significant in the nonparametric McNemar test for paired data (*p* = 0.077).

### 2.3. Transfer Ratio of Aflatoxin B1 to Aflatoxin M1

**Figure 2:**
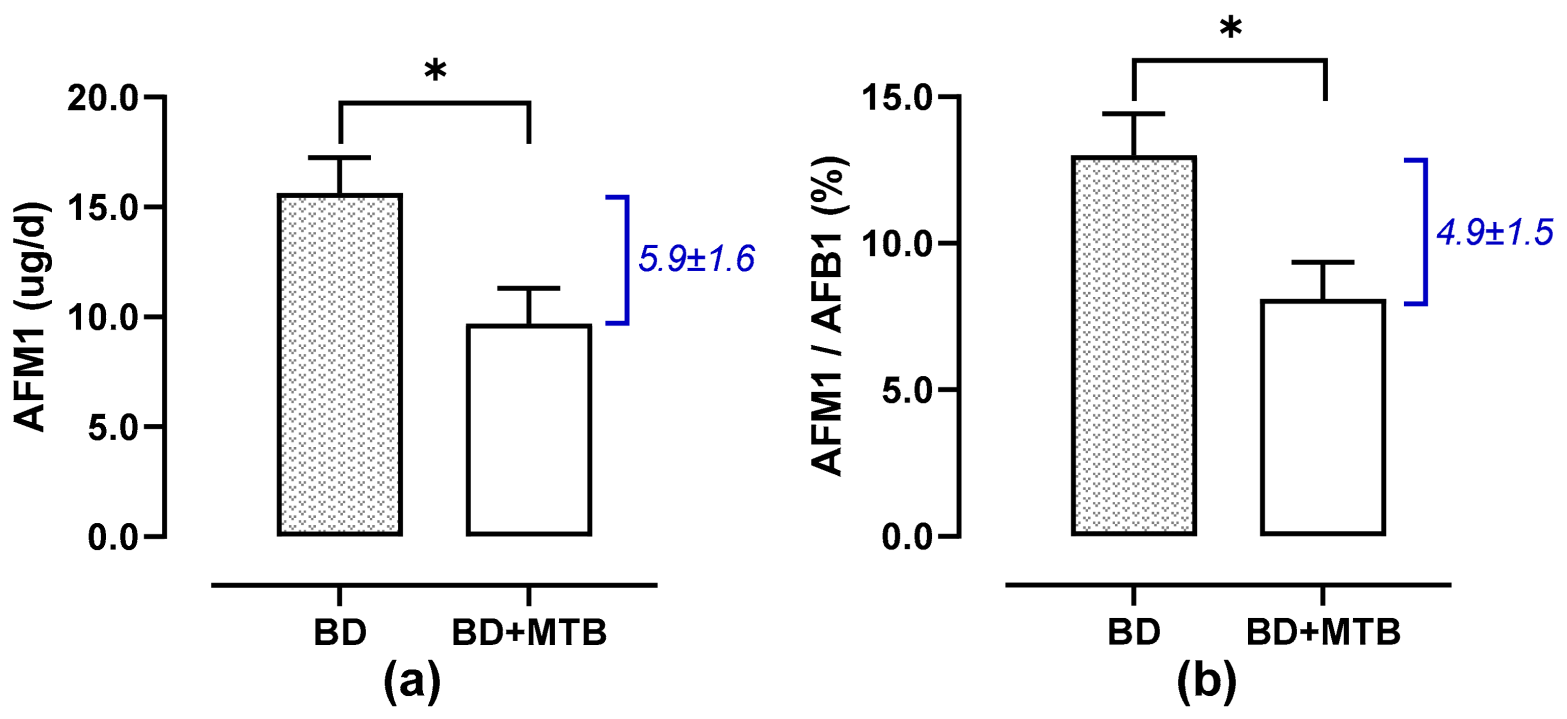
Least squares means (±SEM) of aflatoxin M1 (AFM1) content in milk and the transfer of aflatoxin B1 (AFB1) from the diet to milk in dairy cows fed either a basal diet (BD) or a basal diet plus a mycotoxin binder (BD+MTB). (**a**) Average daily excretion per cow; (**b**) estimated percentage of AFB1 present in the diet (120 µg/d) transferred as AFM1 into milk. The difference between the means (blue text) and the *p*-value of Tukey’s HSD test are also indicated (* *p* < 0.05).

Cows fed the basal diet excreted an average of 15.6 ± 1.7 µg/d of AFM1 in their milk each day (95% CI: 12.3–19.0); however, with the addition of MTB to the feed (Figure 2a), the amount of AFM1 in raw milk showed a significant reduction in the transfer of ingested aflatoxin, resulting in a daily AFM1 excretion of 9.7 ± 1.5 µg/d (95% CI: 6.3–13.1; *p* < 0.05); this resulted in a daily reduction in AFM1 of 5.9 ± 1.6 µg per cow, which can be interpreted as an MTB efficacy of 37.8% in daily AFM1 excretion.

The trial showed that significant differences were observed between the diets (*p*-value < 0.05) in the percentage transfer rate of AFB1 from the feed to AFM1 in the milk (Figure 2b); thus, the transfer rate was estimated at 13.0 ± 1.4% for the basal diet and 8.1 ± 1.3% for the basal diet plus a sequestrant (95% CI: 10.2–15.8 and 5.3–10.9%, respectively). This suggests that the MTB added to the basal diet was 37.7% effective in reducing the average transfer rate of AFB1 from feed to AFM1 in raw milk (*p* < 0.05). Moreover, neither the period alone nor the interaction between the period and the diet had a statistically significant effect (*p* > 0.05) on milk yield, AFM1 concentration in milk, daily AFM1 excretion, or the transfer rate of AFB1 from feed.

## 3. Discussion

In this study, the raw milk was contaminated with AFM1, so an experiment was conducted entirely under dairy farm conditions. The trial suggested that adding a mycotoxin binding agent to the cows’ diet significantly reduced the average concentration of AFM1 in milk, achieving a reduction of 33.8% (0.724 to 0.479 µg/kg; *p* < 0.01). Furthermore, with the addition of the MTB to the basal diet, the transfer rate decreased both the daily amount and the proportion of AFB1 from the feed to AFM1 in raw milk (15.6 to 9.7 µg/d; 13.0 to 8.1%; *p* < 0.05). These average values suggest that the use of the MTB facilitates progress toward the targets voluntarily adopted by Peru’s surveillance programs for MT contamination to meet the recommendations of the Codex Alimentarius [20]. However, the related MCLs vary by region of the world; for example, the European Union legislation includes legally binding regulations and recommendations that guide the achievement of health and economic objectives [19,20,21]. It has been suggested that mycotoxin contamination be prevented during the preharvest phase by inhibiting fungal growth; for this purpose, the use of resistant plants, fungicides, lactic acid bacteria, and non-toxigenic fungal strains has been recommended to competitively reduce toxigenic fungi and MT production [27,28]. Post-harvest strategies have also been proposed to reduce the content and activity of MTs using physical (grain sorting, low moisture and oxygen levels, extrusion, irradiation, cold atmospheric plasma, magnetic materials, etc.) and chemical methods (organic acids, oxidizing agents, polyphenols, natural essential oils, bentonite, chitosan, ozone, nanoparticles, plant extracts, MTBs, etc.) [29,30,31]. In addition to these strategies, key factors include early detection measures in the field and the subsequent use of methods for identifying toxigenic fungi (cultures and molecular assays), as well as chromatographic, immunological, and biosensor techniques for the detection and quantification of MTs [32,33,34]. However, it is recognized that the only way to successfully manage the risk represented by MTs is through a holistic approach that includes hazard analysis and critical control points, as well as good manufacturing practices [2,16]. In this regard, the involvement of farmers and food processors plays a significant role that could be strengthened through field techniques for the timely monitoring of MT contamination.

In this trial, cows were fed a total mixed ration (TMR) naturally contaminated with aflatoxin B1 (2.6 ± 0.5 µg/kg), at a concentration that is considered locally to be below the recommended level (20.0 µg/kg), resulting in an estimated total daily intake of 120 µg of AFB1 per cow. The feed ingredient with the highest contamination level was ground corn (14.2 µg/kg). The feed samples for which AFB1 concentration values were below the LOD were considered not detected and were therefore excluded from the statistical analyses, not because they suggested or implied a total absence of contamination, but rather because they indicated that the analytical instrument was not precise enough to reliably quantify values below 1.0 µg/kg [35]. Although the actual values might have ranged between zero and the LOD, in that case, the same AFB1 concentration to which the group of cows that consumed only the BD was exposed also occurred in the group that consumed the BD plus MTB; therefore, the effects of uncertainty from this variation could be considered balanced between the two groups. For example, if, instead of using the method proposed by Huang et al. [36] of assigning half the LoD to each undetected value, those values are replaced by removing data points below the LoD, then the estimated AFB1 exposure would have decreased by 13.3% (120 versus 104 µg/day) without changing the concentration of AFM1 in milk, so the estimated mean values for the AFB1 transfer rate would increase for both the BD and the BD+MTB diets compared to those previously presented in this study (120 µg/d: 13.0% and 8.1% versus 104 µg/d: 15.1% and 9.3%, respectively), without changing the statistical significance of the difference between the means *(p* < 0.05); furthermore, the efficacy of MTB in reducing the AFB1 transfer rate would also be equal to the previous value (37.7%). However, the substitution of not-detected values assumes that all feed samples had some degree of AFB1 contamination and that the data follow a normal, log-normal or gamma distribution, which may not hold for the dataset obtained [35,36]. If the behavior of AFB1 concentrations below 1.0 µg/kg were to be investigated using an HPLC or LC/MS method with a lower LOD, then the study could no longer be conducted under field conditions [6,33]. Therefore, it was acknowledged that the limitations of the field study also included the small available number of homogeneous animals, which prevented the conduct of a fully randomized study, as well as limited technical and instrumental resources.

The estimated concentration of AFB1 in this study was within the usual range found in dairy production units in Latin America and other parts of the world [15,37,38,39,40]. However, this level of feed contamination can lead to the presence of significant concentrations of AFM1 in milk and therefore should constitute a trade barrier to livestock production [17,18,19,20,21]. Aflatoxin contamination of ground corn or corn silage has already been well documented [31,41,42]. Although the presence of other mycotoxins was not analyzed in the present study, it is possible that dairy feeds could contain other AFs and additional MTs besides AFB1; however, AFB1 contamination has been the most regulated mycotoxin because it is typically the most abundant in dairy farming and poses the greatest risk to public health, as well as to animal health and livestock productivity, even at concentrations as low as 10 µg/kg of feed [4].

The transfer rate observed in this study (8.1–13.0%) was considered slightly elevated but fell within the ranges reported in other studies. Veldman et al. (1992) [43] reported a transfer rate of 1.8–6.2% of AFB1 from feed to AFM1 in cow’s milk; therefore, they suggested that, to prevent milk from exceeding the MCL for AFM1 in the European Region (0.05 µg/kg), the average daily intake of AFB1 should be less than 40 µg/kg of AFB1 in the feed. Likewise, a panel of experts from the European Commission (2004) [18] concluded that the estimated transfer rate (2.0–6.0%) is also related to the amount of feed consumed and that the amount of feed is, in turn, associated with the nutritional requirements derived from milk production; therefore, they estimated that the suggested range of total daily intake could vary between 35 and 265 µg/kg of AFB1 in the feed. More recent studies have shown wider variations in the transfer rate; Bervis et al. (2021) [38] found an average rate of 8.6% but a range of 0.76 to 36.0%, while Zentai et al. (2023) [44] described a range of 0.75 to 6.2% in their review studies.

The statistical analysis of general linear models conducted in this study found that the inclusion of the MTB had a significant effect (*p* < 0.05) on the concentration, total excretion, and transfer rate of AFM1 in raw milk. However, no effect of the test period or its interactions was detected for any of these variables. Unexpectedly, the individual effect of the cows on the percentage transfer rate reached statistical significance (*p* < 0.01); this effect was because two cows identified as “A” and “E” showed higher AFM1 concentration values when they ingested the MTB compared to the basal diet alone. Similarly, some experimental studies have shown that the transfer of AFB1 to AFM1 can increase in animals depending on variations in liver function, health status, energy balance, and lactation stage [8,45,46]. The statistical effect of isolated animals yielding inconsistent results may diminish with larger sample sizes. The design used in this study proved to be efficient because it reduced variability among the animals, since each cow served as her own control by receiving the other diet in the subsequent period; furthermore, a relevant carryover effect was avoided during the washout period, as 99.9 ± 0.02% of the AFB1 administered in the first period was removed before the start of the second period. Thus, the results of this study showed that the statistical power reached 94.4%, estimated under a crossover design with a sample size of 12 animals.

The effect of MTBs has been widely documented in numerous studies, which establish the efficacy of various products in controlled experimental studies, reviews of specific topics related to MTBs, or analyses of their use in specific populations [9,10,22,23,24,25,45,46]. These previous studies have reported results showing that the adsorption capacity of MTBs can vary widely in effectiveness; however, the risk of also sequestering vitamins and minerals from the diet when the optimal dose estimated by the manufacturer is exceeded has been highlighted [25]. Therefore, in the present study, it was deemed appropriate to adhere to the recommended dose.

In this trial, MTB was found to be 37.8% effective in preventing the transfer of AFB1 into milk (15.6 to 9.7 µg/d), while other studies have reported relatively similar values; for example, Cha et al. [47] tested two MTBs in an experiment in which they directly supplied purified aflatoxin to dairy cows, and the reduction in AF concentration by the two MTBs (49.1 and 54.3%) was verified using high-performance liquid chromatography in combination with fluorescence detection. Furthermore, Nishimwe et al. [48] showed the effectiveness of technology transfer for mycotoxin control by providing an MTB (calcium montmorillonite clay) and training in its use to a group of dairy farmers in Rwanda. After a three-month follow-up, it was shown that the mean concentration of AFM1 in milk was significantly lower (50.0%) compared with that in a control group of farms without MTB or training. However, in the present study, the central objective was to validate, in the Peruvian context, a methodology for quantifying aflatoxin contamination using minimal equipment, enabling farmers to directly assess the production process and obtain the information needed to reduce natural mycotoxin contamination in feed and milk before they are delivered for human consumption.

Various methodologies have been used to assess MT contamination in feed and milk; in particular, HPLC methods with a fluorescence detector and liquid chromatography in tandem with mass spectrometry are considered the official reference methods [6,33]. In addition, some rapid, low-cost tests have been successfully implemented, requiring minimal equipment and analytical expertise. For example, Serraino (2019) [34] used the lateral flow immunochromatography technique on more than 30,000 milk samples to estimate the proportion of hepatocellular carcinoma cases attributable to AFM1 exposure in different Italian population groups. The methodology used in this study was a lateral flow immunochromatography assay for the detection of aflatoxins in milk or feed samples. Inside this device, the AF in the sample competes with the anti-AF antibody on the gold microspheres for binding to the test line. The unbound antigen is deposited on the control line, and the reflectance is quantitatively compared to the test line using a reader that determines the concentration of AF in the sample using an algorithm. In some interlaboratory assessments of these devices using artificially contaminated samples, the false-negative and false-positive rates were calculated to be less than 5%, while the average intra-laboratory repeatability and inter-laboratory reproducibility were estimated at 11% and 13%, respectively; therefore, the test has been deemed suitable for assessing contamination in feed and milk prior to feeding cows or delivery to processing plants [49,50,51].

## 4. Conclusions

The study showed that a mycotoxin binder, made from yeast fragments and calcium carbonate, significantly reduced aflatoxin M1 contamination in raw cow’s milk. In this regard, MTB was 33.8% effective in reducing the mean concentration of AFM1 in raw milk (0.72 to 0.48 µg/kg; *p* < 0.01); the daily amount of AFM1 excreted in milk per cow also showed a 37.8% reduction (15.6 to 9.7 µg/d; *p* < 0.05), as well as a 37.7% reduction in the average transfer rate of AFB1 from feed to AFM1 in raw milk (13.0% to 8.1%; *p* < 0.05). Furthermore, cows fed the MTB showed no significant changes in daily milk production.

The experiment and analytical techniques were conducted entirely under field conditions, suggesting that a field-screening evaluation may be relevant for the design and successful implementation of a critical control point program to manage mycotoxin contamination on the dairy farm.

## 5. Materials and Methods

### 5.1. Study Design

The study was conducted at an intensive dairy farm located in the Department of La Libertad, Peru. This tropical region (7°29′30″ S and 79°37′15″ W), situated on the coastal side of the Andes Mountains, is characterized by a dry semi-arid climate (D(i)B’) typical of the northern coastal valley of the country, with an average elevation of 104 m above sea level and low precipitation (15 mm/year). During the trial (February–March 2022), the average ambient temperature ranged from 19.5 to 25.4 °C, and the average relative humidity was 75%.

The participating dairy farm had individual free-stall barns with sand bedding and open sides, as well as partial confinement pens for lactating cows, cows in transition, heifers, and calves. The facilities were equipped with linear/individual feeders, permanent drinkers, and areas for resting, exercise, and shade. Feeding was based on irrigated production of alfalfa, corn, rye grass, and clover, in addition to agro-industrial byproducts and commercial concentrate supplements. There was also a mechanical milking parlor and milk cooling tank, as well as records of production, reproduction, vaccination programs, and mastitis control.

The cows were selected based on similar age, body weight, number of calvings, days in lactation, and the absence of physical abnormalities and clinical signs of disease. The trial was conducted with 12 multiparous Holstein cows, aged 3.2 ± 0.5 years, 220 ± 20 days into lactation, with an average daily milk production of 20.5 ± 1.2 kg/cow, and with a mean body condition score of 2.7 ± 0.2. The selected cows were arranged by the identification number they had received at birth, which was permanently marked on an ear tag. Subsequently, the cows were systematically assigned to one of the two diets during the first period of the trial, whereas, during the second period, the cows received the diet they had not received in the initial period. Based on the toxicokinetic data mentioned previously, each experimental period lasted 6 days (Table 2), and intermediate washout periods (AFB1-free diet) of the same duration were implemented between them. The number of animals included in the trial was considered adequate, as similar sample sizes per treatment had been successfully used in several previous trials with MTB [9,22,38,39].

### 5.2. Experimental Diet

The basal diet was formulated in accordance with the recommendations of the National Research Council (NRC, 2001), based on the nutritional requirements for body weight and milk yield of the cows included in the study. The experiment was conducted using two types of diets: the basal diet (BD) and the control diet (CD); the same BD to which only the mycotoxin binder (MTB) was added at a dose of 150 g/d per cow was designated BD+MTB. Each of the ingredients used in the preparation of the CD was tested using the sampling and quantification methods described below to ensure that they did not contain detectable levels of AFB1. Table 1 shows the daily amount of feed ingredients consumed per cow (kg/day), the mean AFB1 concentration detected in each ingredient (µg/kg), the weighted contribution of the ingredient to the TMR (µg/d), and the total daily AFB1 intake per cow in BD and CD in periods I and II. The animals were fed twice a day (7:00 and 15:00 h) with a TMR using individual feeders; each cow received 40.2 kg of TMR daily, naturally contaminated with AFB1. The protein–energy feed ingredients (ground corn, soybean cake, sugarcane molasses, and whole soybean meal) used in all experimental periods were pre-mixed in the proportions shown in Table 1 and stored in a warehouse until use. The other diet ingredients (alfalfa hay, whole-plant corn, and premix) were mixed daily with the protein–energy mixture before feeding and dispensed using a motorized mixer cart. The MTB was added to the mineral premix; this product consisted of calcium carbonate and yeast fragments (Vitafix^®^, Nuscience Group, Drongen, Belgium); it was purchased on the local market, and the manufacturer was not involved in the study or dissemination of its results.

### 5.3. Quantification of Aflatoxin Levels in Feed and Milk

Prior to the start of the experimental period, each feed ingredient included in the basal and control diets was sampled to quantify the presence of AFB1 following the protocols established in the European Regulation [52], as well as the recommendations included in the Codex Alimentarius sampling plans regarding aflatoxin contamination [20]. Therefore, the feed ingredients were separated in the dairy farm’s storage area in quantities sufficient to prepare the TMR used throughout the experiment. Incremental samples were taken from each ingredient in quantities proportional to the batch size; these samples were then thoroughly homogenized, and a composite sample of 1.0 kg was taken for every 10 incremental samples; lastly, the composite samples were subdivided into 200 g laboratory samples. Before the start of each experimental period, the AFB1 concentration in the TMR of the diets (CD, BD, and BD+MTB) was verified using the same sampling procedure. To quantify aflatoxin levels in feed within the dairy farm facilities, the Rapid One Step Assay Charm lateral flow immunochromatographic system (ROSA; Charm Sciences Inc., Lawrence, MA, USA) was used in accordance with the manufacturer’s instructions. Aflatoxins were extracted from the pulverized samples using methanol (70%) and vigorous shaking. Subsequently, a clarified extract was obtained by centrifugation and filtration. The extract was diluted in a buffer (100 µL/1.0 mL), and 150 µL was applied to the sample pad of the lateral flow device for quantitative testing of AFB1 (ROSA LF-AFQ-FAST, Charm MRL Aflatoxin B1; Charm Sciences Inc., Lawrence, MA, USA). After 5 min in a ROSA incubator (Charm LF-INC4-3-45D), the result was quantified in µg/kg using a ROSA-M reader (Charm LF-ROSA-EZ-M).

The cows were milked twice a day (06:00 and 18:00 h), and each animal’s milk production was recorded daily. On days 5 and 6 of each treatment period, 4 individual milk samples were collected during consecutive milkings (300 mL), and a composite sample was prepared from each cow for quantitative analysis. The milk samples were analyzed on-site using a lateral flow immunochromatographic system with the ROSA Aflatoxin M1 Charm-MRL quantitative test, in accordance with the manufacturer’s instructions (MRLAFMQ, Charm Sciences Inc., Lawrence, MA, USA). The milk sample was shaken, and 300 μL was dispensed directly onto the device’s sample pad; after 15 min of incubation, the result was read on a ROSA-M reader.

All measurements were performed in duplicate in the presence of purified AFB1 and AFM1 standards (Charm Sciences Inc., Lawrence, MA, USA) to verify the detected mycotoxin concentrations. Samples were reanalyzed when the coefficient of variation between replicates exceeded 20%. When the concentration of AFB1 or AFM1 exceeded the detection limit, the assay was repeated using an appropriate dilution with feed or milk containing no detectable levels of aflatoxins. AFB1 concentration values for the ingredients and the BD, CD and BD+MTB diets that were below the LOD were considered not detected [35,36]. They were therefore excluded from the statistical analyses because the analytical instrument was not sufficiently precise to quantify values below the LoD reliably. Calibration curves were prepared using purified standards, and the reference values (range: 1.0–100 and 0.05–0.750 μg/kg; coefficients of variation: CV, 9.7% and 13.2%; and limits of detection: LOD, 1.0 and 0.05 μg/kg) were validated in-house (Table 3); the range, CV, and LoD were estimated following the procedures described by Armbruster and Pry [53]. The LoD was determined as the minimum concentration of AF that could be estimated using the selected analytical procedure and that differed from the apparent concentration of AF (mean plus *Z*-critical value times standard deviation) found when replicates of a blank sample that did not contain AF were analyzed.

### 5.4. Statistical Analysis

The experiment was designed as an orthogonal Latin square of treatments, with two diets (BD, BD+MTB) and two experimental periods (I, II). Data on milk production, AFM1 concentration, daily AFM1 excretion, and transfer rate were analyzed using generalized linear models with R software, version 3.6.1. The statistical model included fixed effects for diet, period, and the diet × period interaction, as well as a random effect for cow. The statistical model used was stated as *Y_ijk_* = *μ* + *D_i_* + *P_j_* + (*D* × *P*)*_ij_* + *Ck* + *ε_ijk_*, where *Y_ijk_* = observed value of the response variable for cow *k*, which received diet *i* during period *j*, *μ* = overall intercept, *D_i_* = fixed effect of diet, *P_j_* = fixed effect of the experimental period, (*D* × *P*)*_ij_* = fixed interaction between diet and period, *C_k_* = random effect of the cow, and *ε_ijk_* = residual error. Results were expressed as least squares means, standard error of the mean, and 95% confidence intervals. Subsequently, Tukey’s HSD test was used to compare differences between the means of the response variables. AFM1 concentrations in milk samples were classified as categorical data (1 = positive, 0 = negative) based on a comparison between the detected AFM1 concentration and the maximum contaminant level (0.5 µg/kg) recommended in the Peruvian context; the proportion of positive samples for each diet was evaluated using McNemar’s nonparametric test for paired data. A *p*-value of ≤0.05 was considered statistically significant for all statistical procedures.

The transfer rate was calculated using the following formula:

$$Transfer\;rate\left(\%\right)=\frac{{AFM}_{1}\left(\frac{\mu g}{kg}\right)\times milk\;yield\left(\frac{kg/d}{cow}\right)}{{AFB}_{1}\;intake\left(\frac{\mu g/d}{cow}\right)}\times 100$$

The trial power was estimated post hoc at 94.4% for rejecting the null hypothesis of equal AFM1 concentrations between the two diets (BD and BD+MTB), over two periods and under a 2 × 2 crossover design for n = 12 cows, *n* − 1 degrees of freedom (*d.f*.), and α = 0.05. The power (*P*) was estimated using the formula *P* = 1 − *(~N Tc* | λ) + *~N Tc* | λ, where *~N* is the approximation to the normal distribution, *Tc* is the critical value for the two-tailed paired t-probability (α = 0.05, *d.f.* = n − 1), and λ is the noncentrality parameter (λ = *d* √ *n*). The standardized effect size (*d*) was calculated as *d* = Δ/*SD*Δ, where Δ is the mean of the intra-cow differences in AFM1 concentration between BD and BD+MTB (Δ = 0.245 µg/kg), which follows a normal distribution (*p* > 0.05, Shapiro–Wilk test), and *SD*Δ is the standard deviation of the differences (0.224 µg/kg). A lack of a relevant carryover effect is assumed (99.9 ± 0.02% elimination of AFB1 from the previous period), and no significant diet × period interaction was observed (*p* > 0.05) [54]. The following formula was used to calculate the concentration of AFB1 that may have been carried over from period I to period II of the experiment:

$$C\left(t\right)=C_{0}\;{\left(\frac{1}{2}\right)}^{t/t_{1/2}}$$

where $C\left(t\right)$ = concentration remaining at time *t*, $C_{0}$ = initial concentration, 1/2 = a half-life, t = elapsed time, $t_{1/2}$ = elimination half-life, $t/t_{1/2}$ = number of half-lives that have occurred [6].

## Figures and Tables

**Table 1:** Mean concentration of aflatoxin B1 (AFB1) detected in feed ingredients and in the basal and control diets.

Ingredient	Daily Amount		Batch (Ton)	Samples (No.)			AFB1	
	(kg)	(%)		Incremental (No)	Aggregate (kg)	Laboratory (No.)	Mean ± SD (µg/kg) *	(µg/d) **
Alfalfa hay	6.0	14.9	3.0	20	2.0	10	ND	3.0
Ground corn ^a^	7.3	18.2	4.0	40	4.0	20	14.2 ± 0.8	104
Soybean cake ^a^	0.5	1.2	1.0	10	2.0	10	ND	0.25
Sugarcane molasses ^a^	1.0	2.5	1.0	10	2.0	10	ND	0.50
Whole soybean meal ^a^	3.5	8.7	2.0	20	2.0	10	ND	1.75
Whole-plant corn	20.5	51.0	10.0	40	4.0	20	ND	10.3
*** Premix	1.4	3.5	0.8	10	2.0	10	ND	0.70
Basal diet (period I)	40.2	100	3.0	20	2	10	2.58 ± 0.41	120
Basal diet (period II)	40.2	100	3.0	20	2	10	2.59 ± 0.68	120
Control diet (Washout periods)	40.2	100	9.0	60	6	30	ND	ND

^a^ The amount of these protein–energy ingredients used in all experimental periods was premixed and stored until use. The remaining ingredients were incorporated daily as a total mixed ration. * ND = Not detected, below the limit of detection (LoD; < 1.0 µg/kg). ** For the statistical analysis, the ND values for AFB1 concentration (µg/kg) were adjusted to half the LoD value, weighted by the daily intake of AFB1 (µg/d) in each ingredient of the diet. *** Salt, bicarbonate, calcium, and monensin sodium.

**Table 2:** Latin square orthogonal treatment design, with a basal diet (BD) and another BD to which a mycotoxin binder (MTB) was added (BD+MTB). The total mixed rations were fed during two consecutive periods (6 days each), separated by washout periods, with a control diet (CD) containing no detectable levels of aflatoxins ^a^.

COW	Washout Period	Period I	Washout Period	Period II	Washout Period
A	CD	BD+MTB	CD	BD	CD
B	CD	BD+MTB	CD	BD	CD
C	CD	BD+MTB	CD	BD	CD
D	CD	BD+MTB	CD	BD	CD
E	CD	BD+MTB	CD	BD	CD
F	CD	BD+MTB	CD	BD	CD
G	CD	BD	CD	BD+MTB	CD
H	CD	BD	CD	BD+MTB	CD
I	CD	BD	CD	BD+MTB	CD
J	CD	BD	CD	BD+MTB	CD
K	CD	BD	CD	BD+MTB	CD
L	CD	BD	CD	BD+MTB	CD

^a^ BD = Basal Diet naturally contaminated with aflatoxins (AFB1 120 µg/d). BD+MTB = BD plus an MTB (calcium carbonate and yeast fragments, 150 g/d per cow). CD = Control diet with no detectable levels of aflatoxins (<1.0 µg/kg).

**Table 3:** Calibration curves of aflatoxins AFB1 and AFM1 for the internal validation of the limits of detection and quantification (LoD and LoQ) compared to the apparent concentration of the blank samples ^a^.

Aflatoxins (μg/kg) ^b^	Mean	STD	Min	Max	CV (%)	LoB	LoD	LoQ
AFB1						0.02	1.03	2.05
0.00	0.0	0.0	37.6	0.00	0.02			
5.0	4.9	0.6	12.5	3.90	5.5			
20.0	23.1	5.7	24.8	11.8	32.3			
100	93.9	16.1	17.2	62.5	114			
AFM1						0.020	0.032	0.044
0.000	0.013	0.004	29.7	0.008	0.019			
0.025	0.032	0.007	23.4	0.021	0.039			
0.050	0.046	0.010	21.6	0.031	0.060			
0.750	0.723	0.260	36.1	0.559	0.969			

^a^ LoB: Mean of blank + Z (critical value: 1.645) × STD of blank. LoD: LoB + Z × STD of lowest concentration. LoQ: LoD + Z × STD of lowest concentration. ^b^ Number of samples per level = 5.

## Data Availability

The original contributions presented in this study are included in the article. Further inquiries can be directed to the corresponding author.

## Abbreviations

The following abbreviations are used in this manuscript:

AF	Aflatoxin
AFB1	Aflatoxin B1
AFM1	Aflatoxin M1
BD	Basal Diet
CD	Control Diet
FAO	Food and Agriculture Organization
LoD	Limit of Detection
MCL	Maximum Contaminant Limit
MT	Mycotoxin
MTB	Mycotoxin Binder
NRC	National Research Council
ROSA	Rapid One Step Assay
TMR	Total Mixed Ration
95% CI	95% Confidence Interval
